# The Sufferings of Christ

**Published:** 1887-09-10

**Authors:** 


### Words of Consolation.

[Communications for this column should be addressed, "Chaplain," care of the Editor of The Hospital, who will gratefully welcome any suggestions or inquiries from matrons, nurses, and the staff generally.]

### XLIX.—The Sufferings of Christ.

(*For reading to the Sick.*)

It is a dreadful thing to suffer quite alone. However much pain we have to endure, it is an intense relief to have some one within call. Jesus felt the need of companionship in His Sufferings just as much as we do. So He begged the three disciples who accompanied Him into the garden to stay near Him and watch; for His soul was exceeding sorrowful even unto death. They watched awhile, not long enough to give Him the sympathy He wanted, but enough to discern that He began to be sorrowful and to be very heavy. It was not only what He cried aloud in prayer made them think this. They beheld in His poor quivering agitated frame the symptoms of a struggle beyond endurance. They saw Him overcome with a horrible dread, with a tumult of emotion, which laid Him prostrate on the earth. The English translation of the expressions used by the Evangelists to describe the Agony is not nearly adequate. The prostration, the rising from His knees only to fall upon them again several times, the pleading "Abba Father, if it be possible, let this cup pass from Me," amid convulsive sobs, with perspiration pouring like great drops of blood from His forehead to the ground,—all this summed up in the word "Agony," which means wrestling as in a contest, points not only to the terrible ordeal He passed in completely resigning the human will to the will of the Father, but as well, we must believe, to something more dreadful in the nature of sin than has yet been revealed to us. It may help you somewhat to know that He bore the burden of sin and felt the weight as no other could, because He was innocent. It may be further profitable to reflect, that as the Sin-bearer He was given to see sin *as it is*. He must have seen the naked, undisguised wickedness of sin, as the utmost disloyalty to the God it has injured, and whose eternally sacred law and order has been violated by it. He saw it with all its past, present, and future consequences revealed to the eye of His soul, knowing how many sinners for whom He was about to die would refuse the benefits of His sacrifice. If we could see sin as God does we should fathom the Agony and its causes more easily. At least let us never smile again at what plunged Our Saviour into such depths of sorrow. We have most of us shut our eyes to the sinfulness of sin, even if we have never with the fool of the Proverbs "made a mock at it." By meditating upon the Agony we may learn to hate it more, to shrink from its approaches, to dread its dominion. Read your past by the light of Gethsemane, and you will get rid of the mistaken idea (if you ever had it) that because Jesus endured so much, therefore sin need not be any burden to you. There can be no laying upon Him a burden you have never felt the weight of yourself. Our Lord did not drink that cup of bitterness to excuse any of us from being troubled on account of sin. Nay rather, we are admitted to the garden, that we may learn by prayer and watchfulness with Him to feel the pressure of guilt, ere we can fervently desire to have that guilt removed.

(*To be continued.*)

